# Using Evolutionary Conserved Modules in Gene Networks as a Strategy to Leverage High Throughput Gene Expression Queries

**DOI:** 10.1371/journal.pone.0012525

**Published:** 2010-09-02

**Authors:** Jeanne M. Serb, Megan C. Orr, M. Heather West Greenlee

**Affiliations:** 1 Department of Ecology, Evolution and Organismal Biology, Iowa State University, Ames, Iowa, United States of America; 2 Department of Statistics, Iowa State University, Ames, Iowa, United States of America; 3 Bioinformatics and Computational Biology Program, Department of Biomedical Sciences, Iowa State University, Ames, Iowa, United States of America; 4 Bioinformatics and Computational Biology Program, Iowa State University, Ames, Iowa, United States of America; Cairo University, Egypt

## Abstract

**Background:**

Large-scale gene expression studies have not yielded the expected insight into genetic networks that control complex processes. These anticipated discoveries have been limited not by technology, but by a lack of effective strategies to investigate the data in a manageable and meaningful way. Previous work suggests that using a pre-determined seed-network of gene relationships to query large-scale expression datasets is an effective way to generate candidate genes for further study and network expansion or enrichment. Based on the evolutionary conservation of gene relationships, we test the hypothesis that a seed network derived from studies of retinal cell determination in the fly, *Drosophila melanogaster*, will be an effective way to identify novel candidate genes for their role in mouse retinal development.

**Methodology/Principal Findings:**

Our results demonstrate that a number of gene relationships regulating retinal cell differentiation in the fly are identifiable as pairwise correlations between genes from developing mouse retina. In addition, we demonstrate that our extracted seed-network of correlated mouse genes is an effective tool for querying datasets and provides a context to generate hypotheses. Our query identified 46 genes correlated with our extracted seed-network members. Approximately 54% of these candidates had been previously linked to the developing brain and 33% had been previously linked to the developing retina. Five of six candidate genes investigated further were validated by experiments examining spatial and temporal protein expression in the developing retina.

**Conclusions/Significance:**

We present an effective strategy for pursuing a systems biology approach that utilizes an evolutionary comparative framework between two model organisms, fly and mouse. Future implementation of this strategy will be useful to determine the extent of network conservation, not just gene conservation, between species and will facilitate the use of prior biological knowledge to develop rational systems-based hypotheses.

## Introduction

The emergence of system-wide approaches (‘-omics’; e.g., genomics, proteomics, metabolomics, etc.) and related technologies to quantify molecular changes that accompany biological processes or disease states has resulted in an explosion in the amount of data collected by researchers. Investigators across all areas of biology have designed large scale experiments to capture a broader systems-based understanding of gene or protein expression changes that accompany their process of interest. However, many have found that such datasets are too large to be immediately informative, and extracting useful information from these datasets is dependent upon additional analysis.

One strategy to analyze such data is to generate gene network models using one of several analytical frameworks [1]–[5]. In theory, these network approaches have two advantages: they should accelerate the rate of novel discoveries by automating data analysis and they should be more immune to experimenter bias. This use of computational strategies will potentially lead to discoveries from omics data without *a priori* knowledge of the system. However, these computational approaches require a tremendous amount of biological data. For example, if an investigator wants to understand which genes function together during a particular developmental process, she might profile changes in gene expression over developmental time. Ideally the number of conditions (e.g., ages, experimental perturbations) under which gene expression is measured should be much larger than the number of genes being profiled in order to obtain an accurate estimate of the covariance matrix upon which the network of all genes is based [6]. Thus, for a microarray experiment that measures the expression of 5000 genes, one should measure the expression of each gene under more than 5000 different conditions. Even collection of 20% of the ideal amount of data for robust analyses is both time and cost prohibitive for most investigators. As a consequence, the majority of biologists collect datasets that are too small for effective computational analysis and too large for systematic and efficient consideration of candidate molecules. This data limbo is a limiting factor to the growth of the field of systems biology.

While it is essential that the development of computational tools and approaches continue, it is also essential that efforts are made to establish ‘biological heuristics’ that will allow benchtop investigators to perform meaningful analyses on the sometimes limited amounts of data they are capable of collecting. A key first step in this process is to consider the development of strategies to efficiently query omics data, as opposed to exhaustively analyzing it. The use of biological heuristics is a flexible strategy, which utilizes prior biological knowledge of the system to design queries. These queries ask specific questions about relatively small groups of interacting genes and return manageable numbers of candidate genes for further analysis at the bench.

Our approach to querying high-throughput data utilizes prior biological knowledge by starting with a ‘seed-network’ of genes, and is based on the paradigm that the expression of genes that function together will change in similar ways over time (i.e., their expression will be correlated). The basic assumption is that if a gene is correlated with one member of the seed network, it may be involved in the process of interest; however, if the same gene is correlated with multiple members of the seed-network it much more likely to be involved in that process (e.g., retinal cell fate determination). One of us has demonstrated previous success identifying gene candidates in development of rod photoreceptors by using a seed-network-based heuristic to query high throughput data [7], and this success motivated our efforts to further develop strategies to identify effective seed networks to query large datasets.

Here we employ our seed-network approach to a genetic comparison of two important models in the study of retinal development: the fly, *Drosophila melanogaster*, and the mouse, *Mus musculus*. Despite the morphological and developmental disparity of the fly compound eye [8], [9] and the mouse camera-type eye [10], [11], gene conservation during both fly and mouse retinal development is well-documented [12]–[16] and there is an implicit assumption of gene regulatory network conservation as well [17], [18]. However the networks are not completely congruent [19]. We test the hypothesis that gene relationships established in the developing fly retina can be identified in correlation networks generated using gene expression data from the developing mouse retina. Further, we hypothesize that the resulting mouse network will be an effective tool to discover candidate genes and gene networks that function during mammalian retinal development. In this report, we take advantage of two biological systems by constructing a ‘comparative seed-network’ based on studies of retinal determination in fly and use it to query gene expression data from the developing mouse retina. Our study was guided by three objectives: 1) to construct a literature-based seed network representing the relationships between genes involved in retinal determination in the fly; 2) to determine whether the network relationships of fly genes are identifiable among homologous mouse genes in expression correlation networks generated from the developing mouse retina; and 3) to assess whether this strategy, based on evolutionary comparison between model organisms, is a useful method to identify biologically relevant candidate genes important in retinal determination. Based on these objectives, our results demonstrate successful application of this strategy within our experimental system and provide a clear framework to evaluate this approach in other biological areas.

## Results

### Seed network construction in fly

**Figure 1:**
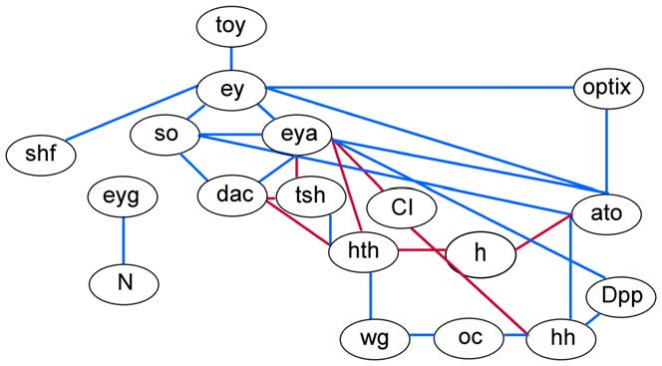
The fly seed network based on experimental results in the literature. Positive correlations between genes are represented by blue edges and negative correlations are represented by red edges. Full names and their abbreviations for *Drosophila* genes are provided in Table 1.

Seed networks are graphs that represent relationships among genes during a biological process, such as retinal determination. These relationships may be physical interactions or causal relations by direct or indirect means, and are represented as edges in the graph or connections (links) in the gene network. We used the results of published experimental studies on eye differentiation in fly to identify a set of 18 genes implicated in fly retinal development, which was built off of the fly retinal determination gene network (RDGN) [12]. We integrated these data into a comprehensive fly ‘seed network’ (Figure 1) based on the work described in File S1.

### Extraction of homologous seed network from mouse datasets

To determine whether the gene relationships represented in the fly seed network are represented in the developing mouse retina, we first converted the literature-based fly seed network into a mouse gene network of putative homologs (Table 1). Then we used BioNet Workbench [http://bionetworkbench.sourceforge.net/] to query four previously published gene expression datasets (I–IV) from mouse [20]–[23]. The datasets were queried for pairwise correlations of >|0.65| between all mouse genes that are homologous to fly seed network members (“seed genes”). A summary of the seed genes and their pairwise correlation values (if >|0.65|) in each of the mouse datasets are given in Table 2. The result was a mouse seed network “extracted” from published gene expression datasets for mouse.

**Table 1:** Fly genes from the seed network and their putative mouse homologs.

Fly gene	Mouse homologs
*toy* (*twin of eyeless*)	*Pax6*
*ey* (*eyeless*)	*Pax6*
*so* (*sine oculis*)	*Six1*/*Six2*
*eya* (*eyes absent*)	*Eya1*/*Eya2*/*Eya3*
*dac* (*dachshund*)	*Dach1*
*Dpp* (*decapentaplegic*)	*Bmp4*
*tsh* (*teashirt*)	*Sdccag33 z*
*hth* (*homothorax*)	*Meis2* (*Meis homoeobox 2*)
*hh* (*hedgehog*)	*Shh* (*Sonic hedgehog*)
*N* (*notch*)	*Notch1*
*wg* (*wingless*)	*Wnt4*
*optix*	*Six3*/*Six6*
*ato* (*atonal*)	*Atoh7* (*Atonal7*)
*h* (*hairy*)	*Hes1*
*eyg* (*eye gone*)	*Pax6*(*5a*)
*CI* (*Cubitus interruptus*)	*Gli1* (*GLI-Kruppel* family member)
*oc* (*ocelliless*)	*Otx1*
*shf* (*shifted*)	*Wif1* (*Wnt inhibitory factor 1*)

**Table 2:** Correlation of network seed genes in each of the four expression datasets of mouse.

Mouse network seed genes	I	II	III	IV
*Pax6*	-	*Notch1* (0.891)	*Tshz1* (1.0)	*Notch1* (0.786), *Six3* (0.762), *Six6* (0.667), *Gli1* (0.810)
*Six1*	NA	NA	NA	-
*Six2*	NA	NA	NA	*Eya1* (−0.667), *Bmp4* (−0.714)
*Eya1*	NA	NA	-	*Six2* (−0.667), *Bmp4* (0.905), *Notch1* (−0.762), *Six3* (−0.690)
*Eya2*	*Atoh7* (−0.826)	NA	NA	*Eya3* (0.761), *Dach1* (0.667), *Six6* (0.738)
*Eya3*	-	NA	NA	*Eya2* (0.761), *Dach1* (0.761), *Wnt4* (0.667)
*Dach1*	NA	NA	NA	*Eya2* (0.66), *Eya3* (0.762)
*Bmp2*	NA	NA	NA	*Gli2* (0.66)
*Bmp4*	NA	NA	*Hes1* (1.0)	*Six2* (−0.714), *Eya1* (0.905), *Notch1* (−0.810)
*Tshz1*	NA	NA	*Pax6* (1.0)	-
*Meis2*	NA	NA	NA	NA
*Shh*	NA	NA	NA	*Six6* (−0.66), *Gli1* (−0.69), *Atoh7* (−0.922)
*Notch1*	NA	*Pax6* (0.891)	*Six3* (0.9)	*Pax6* (0.786), *Eya1* (−0.762), *Bmp4* (−0.810), *Six3* (0.881), *Six6* (0.881)
*Wnt/wnt4*	NA	NA	NA	*Eya3* (0.667), *Gli2* (−0.667)
*Six3*	*Hes1* (0.706), *Gli1* (0.923), *Atoh7* (0.690)	-	*Notch1* (0.9)	*Pax6* (0.762), *Eya1* (−0.690), *Six6* (0.833), *Notch1* (0.881), *Gli1* (0.667)
*Six6*	*Hes1* (0.696), *Atoh7* (0.658)	-	NA	*Pax6* (0.667), *Eya2* (0.738), *Shh* (−0.66), *Six3* (0.833), *Notch1* (0.881)
*Atoh7*	*Eya2* (0.825), *Hes1* (0.799), *Six3* (0.690), *Six6* (0.658)	-	NA	*Bmp2* (0.731), *Shh* (−0.922)
*Hes1*	*Gli1* (0.705), *Six3* (0.706), *Six6* (0.696), *Atoh7* (0.799)	NA	*Bmp4* (1.0)	NA
*Gli1*	*Hes1* (0.705), *Gli1* (0.924)	NA	NA	*Pax6* (0.810), *Shh* (−0.69), *Six3* (0.667)
*Gli2*	NA	NA	NA	*Bmp2* (0.66), *Wnt4* (−0.667)
*Otx1*	NA	-	NA	NA
*Wif1*	NA	NA	NA	NA

The mouse expression datasets are: I [20]; II [21]; III [22], IV [23]. Numbers in parentheses are the positive or negative correlation coefficient of seed genes in each mouse datasets. “-” indicates that the seed gene is present in the dataset, but is not correlated with other seed genes. “NA” indicates that the seed gene is not present in the dataset.

Based on the finding that a subset of relationships from the fly seed network appear to be conserved in the developing mouse retina, we hypothesized that the extracted seed-network (ESN) of mouse gene relationships would be useful for querying the mouse gene expression data to identify additional candidate gene network members. To identify candidates, genes correlated >|0.65| with each gene in the extracted seed-network were retrieved from each dataset and the lists were compiled. Lists of genes correlated with each ESN gene were analyzed to identify genes that correlated with more than one gene in the ESN. Based on the paradigm that genes correlated with multiple ESN genes are likely to have a functional relationship to the gene network, we focused our analysis on 46 candidate genes that were correlated with three or more ESN members (Table 3). Among these 46, 39 genes were correlated minimally with *Eya1*, *Notch1* and *Six3*. We evaluated the relevance of candidate genes identified by this comparative seed-network approach in three ways.

**Table 3:** List of 46 candidate genes found in three or more seed gene lists.

Candidate gene	Description	Function	Reported links	Fly Homolog
*Aplp2* a	Amyloid precursor protein family member in the Alzheimer's disease amyloid beta protein superfamily	Embryonic development of several brain regions [35], [45], [86]	***CNS development	*β amyloid protein precursor-like* (*Appl*)
*Blvra* b	*biliverdin reductase A*	Heme catabolic process [42], [69]	Brain development	None; Only identified in vertebrates
*Bola2* b	Stress-induced morphoprotein, *BolA* type superfamily	Involved in cell proliferation or cell-cycle regulation [51]	Development	CG33672
*Capn2* c	*calpain 2*	Calcium-activated neutral proteases; blastocyst development [29], [50], [66], [74]	Retina	*CalpA-RB*
*cdkn1c* ( = *CDKI*, *Kip2*, *p57Kip2*)^b^	*cyclin-dependent kinase inhibitor 1C* family	Cell cycle arrest [39], [40], [65], [77]	Retina/CNS development	*dap* (*dacapo*)CG1772
*crmp1* b	*collapsin response mediator protein 1*	Hydrolase activity [34], [82]	Retina/Brain development	*CRMP*
*dpysl4* ( = *CRMP-3*, *Crmp3*, *DPY4*, *Drp-4*, *Ulip4*, *unc-33*-*like phosphoprotein 4*)^b^	dihydropyrimidinase-like 4 in the cyclic amidohydrolases protein superfamily	Plays a role in dendrite arborization, guide-posts navigation, and neuronal plasticity [46], [70], [71], [83]	Brain development	*CRMP*
*dynlt1b* ( = *Dynlt1*, *Tctex-1*, *Tctex1*)^b^	dynein light chain, Tctex-type protein superfamily	Microtubule-based processes [54], [85]	Brain development	*Dlc90F*
*ebf1* ( = *O/E-1*, *Olf-1*, *Olf1*)^b^	early B-cell factor 1 in the transcription factor, collier type protein superfamily	Multicellular organismal development; positive regulation of transcription [37], [59]	Retina/Brain development	*kn* (*knot*)
*Ephb2* b	*Eph receptor B2*, part of *tyrosine-protein kinase* protein superfamily	Axon guidance in RGC [30]	**/***Retina development	*eph receptor tyrosine kinase* (*eph*)
*fabp5* ( = *E-FABP*)^b^	*fatty acid binding protein 5*, epidermal	Expressed in neurons during axonal growth in development and nerve regeneration; involved in RGC differentiation and axon growth [26], [58]	Retina/Brain development	CG6783
*Fyn* ( = *SLK*, *SYN*)^b^	Protein tyrosine kinase	Involved in axon guidance; RGC targeting in the Superior colliculus [57]	***Retina/Brain development	*Btk family kinase at 29A* (*Btk29A*); CG8049
*gng5* ( = *G(y)5*, *Ggamma5*)^d^	guanine nucleotide binding protein (G protein), gamma 5	G-protein coupled receptor protein signaling pathway; expressed in precursor cells during neurogenesis [20], [28]	Retina/Brain development	*G protein γ 1* (*Ggamma1*)
*gstm5* ( = *GST*)^b^	*glutathione S-transferase*, *mu 5*	Transferase activity; Expressed in developing lens and retina and may play a role in apoptosis suppression [24], [25]	Retina	Probably distantly related to *glutathione S transferase S1* (*GstS1*); no *mu* homolog in *Drosophila*
*Hsf1* b	*Heat shock factor 1*	Expressed in RGC [48]	Retina	*Heat shock factor* (*Hsf*)
*isoc1* b	*isochorismatase domain containing 1*	Metabolic processes; expressed in medulla oblongata of postnatal adult [68]	Brain development	CG3663, CG11333
*kcnab2* ( = *Kvbeta2*)^b^	potassium voltage-gated channel, shaker-related subfamily, beta member 2	Modulates action potential propagation and neurotransmitter release in hippocampal formation [63]	Brain development	*Hook* (*Hk*)
*krtcap2* b	keratinocyte associated protein 2	Protein amino acid N-linked glycosylation via asparagine [20]	Retina/CNS development	CG31460
*lsm3* b	LSM3 homolog, U6 small nuclear RNA associated (*S. cerevisiae*)	mRNA processing, nuclear mRNA splicing, via spliceosome [60]	Retina/CNS development	CG31184
*mapre1* b ( = *Eb1*)	microtubule-associated protein, RP/EB family, member 1	Kvbeta2 axonal targeting depends on its ability to associate with the microtubule plus-end tracking protein EB1 [44], [75]	Brain development	*Eb1*, CG15306, CG32371, CG18190, CG40354, CG31907, CG2955
*Ndn* e		Required for development of GnRH secreting neurons [61]	***Brain Development	None
*nme2* ( = *nm23-M2*)^b^	non-metastatic cells 2, protein (NM23B) expressed in	mRNA levels increased during retinal degeneration [27], [49]	Retina/Brain development	*abnormal wing discs* (*awd*)
*nsmce1* b	non-SMC element 1 homolog (*S. cerevisiae*)	DNA recombination and repair	NA; No papers found in Pubmed under nsmce1	CG11329
*Pafah1b3* b	platelet-activating factor acetylhydrolase, isoform 1b, alpha1 subunit	May play a role in neuronal migration (based on identified human mutations associated with brain malformation) [79]	Brain development	*Platelet-activating factor acetylhydrolase alpha* (*Paf-Ahalpha*)
*Pcyt2* d	phosphate cytidylyltransferase 2, ethanolamine	Biosynthesis of ethanolamine phospholipids. KO of pcyt2 embryonic lethal [43]	Development	*Phosphoethanolamine cytidylyltransferase* (*Pect*)
*prps1* f	phosphoribosyl pyrophosphate synthetase 1	X-linked enzyme mediates the biochemical step critical for purine metabolism and nucleotide biosynthesis; loss of function associated with optic atrophy [38], [52]	Retina	CG6767
*prrt1* b	proline-rich transmembrane protein 1	Expressed in mouse retina [108]	Retina	None; homologs only in vertebrates
*psme1* ( = *PA28a*)^b^	proteasome (prosome, macropain) 28 subunit, alpha	Component in the ubiquitin-proteasome system that may play an important role in neuronal apoptosis [41]	Brain	*REG*
*Rac1* b	Small gtp aseRAS-related C3 botulinum substrate 1	Involved in actin cytoskeleton regulation; expressed in developing mouse retina, involved in RGC axon behavior; essential for brain development [36], [62], [81]	***Retina/Brain development	*Rac1*, *Rac2*
*rpl10* b	*ribosomal protein 10*	Translation	NA	*Qm*
*rpl27a* g	*ribosomal protein L37*	Expression decreases during maturation of cultured human fetal astrocytes [56]	Brain development	*RpL27A*
*rpl37* b	*rbosomal protein 37*	Dimorphic expression in developing zebra finch brain [80]	Brain development	*RpL37A*, *RpL37B*
*rps11* b	*ribosomal protein S11*	Translation	NA	*RpS11*
*rps26* b	*ribosomal protein S26*	Translation	NA	*RpS26*
*rps3* h	*ribosomal protein S3*	Neuroprotective effect in the brain (hippocampus) exposed to ischemia [47]	Brain	*RpS3*
*rps5* b	*ribosomal protein S5*	Translation	NA	*RpS5a*, *RpS5b*
*Snrpe* b	*small nuclear ribonucleoprotein E*	mRNA processing; expressed in mouse eye and brain on embryonic day 13.5 and postnatal day 0 [60]	Retina/CNS development	CG18591
*Snrpg* ( = *SMG*)^b^	*small nuclear ribonucleoprotein polypeptide G*	mRNA processing; expressed in mouse eye and brain on embryonic day 13.5 and postnatal day 0 [60]	Retina/CNS development	*Small ribonucleoprotein G* (*SmG*)
*Stmn2* ( = *SCG10*)^b^	*stathmin-like 2 in Op18/stathmin* protein superfamily	Microtubule destabilization; RGC growth and cone behavior; expression in mature RGCs and amacrine cells in rat retina [67], [78]	Retina development	*stathmin* (*stai*); CG11298
*tex261* ( = *TEG*-*261*)^d^	*testis expressed gene 261*	Positive regulation of apoptosis	NA	CG3500
*tmsb10* ( = *Ptmb10*, *TB10*)^h^	thymosin, beta 10	Actin cytoskeleton organization; involved in the dynamics of actin polymerization during migration and extension of neurons in the cerebellum [31], [33]	Brain/CNS development	*Ciboulot* (*cib*)
*tor2a* b	torsin family 2, member A	TOR2A mRNA expression and is spliced into preprosalusin; Salusin-beta stimulates the release of arginine-vasopressin from rat pituitary [76]	Brain	*torp4a*
*Txn1* b	*Thioredoxin 1*	Redox activity; expressed by RGC; protective against oxidative insult [64]	Retina	*Thioredoxin2* (*trx-2*); *Thioredoxin T* (*TrxT*); CG13473; *deadhead* (*dhd*)
*unc13b* ( = *Munc13-2*, *Unc13h2*)^a^	*unc-13 homolog B* (*C. elegans*)	Responsible for vesicle priming in glutamatergic nerve cell and gamma-aminobutyratergic (GABAergic) synapses of the hippocampus [72], [84]	Brain	*unc-13*
*wdr78* a	*WD repeat domain 78*	Expressed in mouse eye during embryonic day 12.5,13.5, and 14.5 and mouse retina [108]	Retina	CG7051; CG13930
*Zic2* b	*Zinc finger protein of cerebellum 2*	DNA binding; guidance of RGC axons (*Zic2* expressed by ipsilateral projecting neurons-by inducing expression of *ephB1*); important for forebrain formation; shown to interact with *Gli* proteins [53], [55], [73]	***Retina/Brain Development	*Odd-paired* (*opa*)

Groups of correlated seed genes are given a letter designation:

^**a**^ *Six3*, *Notch1*, *Tshz1*;

^**b**^ *Six3*, *Eya1*, *Notch1*;

^**c**^ *Eya1*, *Notch1*, *Bmp4*;

^**d**^ *Six3*, *Eya1*, *Notch1*, *Bmp4*;

^**e**^ *Six3*, *Notch1*, *Dach1*;

^**f**^ *Eya2*, *Eya3*, *Notch1*, *Tshz1*;

^**g**^ *Eya1*, *Notch1*, *Tshz1*;

^**h**^ *Six3*, *Eya1*, *Notch1*, *Pax6*.

Candidate gene synonymies are provided in parentheses. “RGC” are retinal ganglion cells. “NA” indicates no previous report of the candidate gene specifically involved in retina, retinal development, CNS development, brain development, or development as searched in PubMed.

*CRMP3 is a direct target of calpain that cleaves CRMP-3 at the N terminus [46].

**Biological Process GO Annotation of Neural Retina Development (0003407).

***Biological Process GO Annotation of Nervous System Development (0007399).

First, we performed a manual literature search to find reports of candidate genes' association with the retina, retinal development, brain development or other developmental processes. Results from this manual search are given in Table 3. Forty out of 46 (86%) candidate genes have been previously reported to be associated with one or more of these topics [20], [24]–[86]. Additionally, eight candidate genes (17%) are associated with retinal ganglion cells (RGCs) or RGC development in previous experimental studies [26], [30], [36], [48], [53], [55], [58], [62], [64], [67], [73], [78], [81] (Table 3).

**Figure 2:**
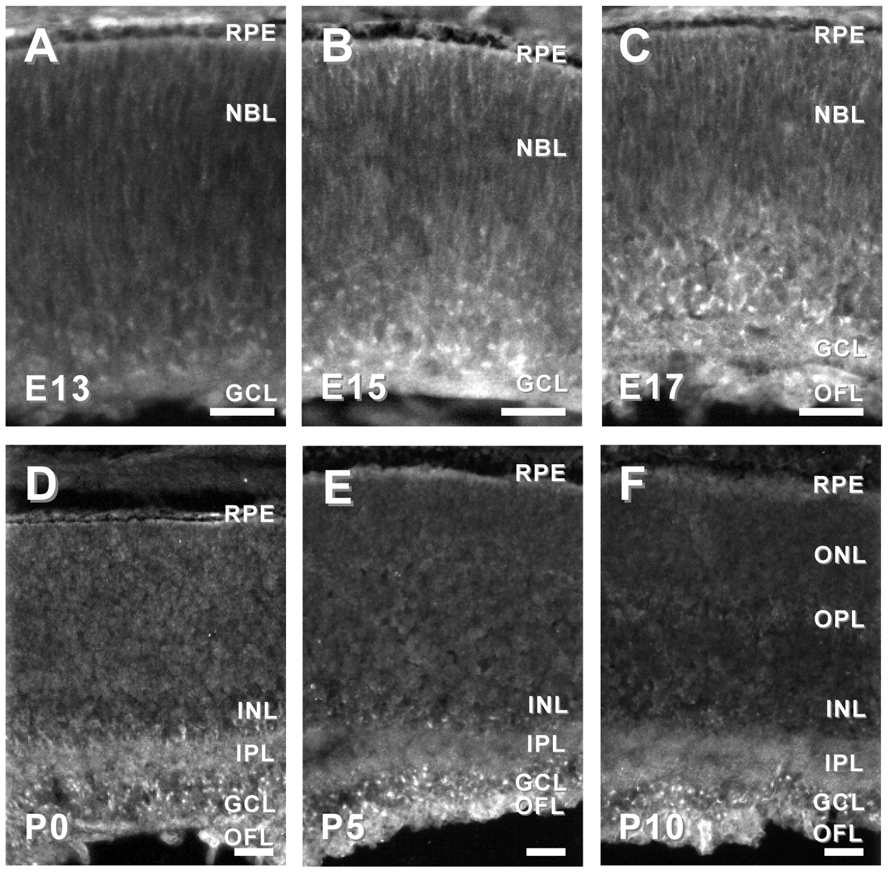
Dynamic protein expression of APLP2 in developing mouse retina. APLP2-IR in the E13 mouse retina was slightly more intense in cells in the inner and outermost retina (A). In the E15 mouse retina (B), APLP2-IR was observed throughout the thickness of the retina, though the most intense immunoreactivity remained localized to cells in the in the inner and outermost retina. By E17, APLP2-IR was largely restricted to cells and processes in the inner one-third of the retina (C). By the day of birth (P0), APLP2-IR was restricted to cell bodies in the ganglion cell layer (GCL), the IPL and the inner nuclear layer (INL). In the P5 retina, APLP2-IR was most prominent in the IPL, GCL and OFL, though some punctate APLP2-IR remained in the INL (D). By P10, APLP2-IR was further restricted to the IPL and OFL, with punctate immunoreactivity only present in the GCL. Abbreviations: GCL, ganglion cell layer; INL, inner nuclear layer; IPL, inner plexiform layer; NBL, neuroblastic layer; OFL, optic fiber layer; ONL, outer nuclear layer; RPE, retinal pigment epithelium. Bars, 30 µm.

**Figure 3:**
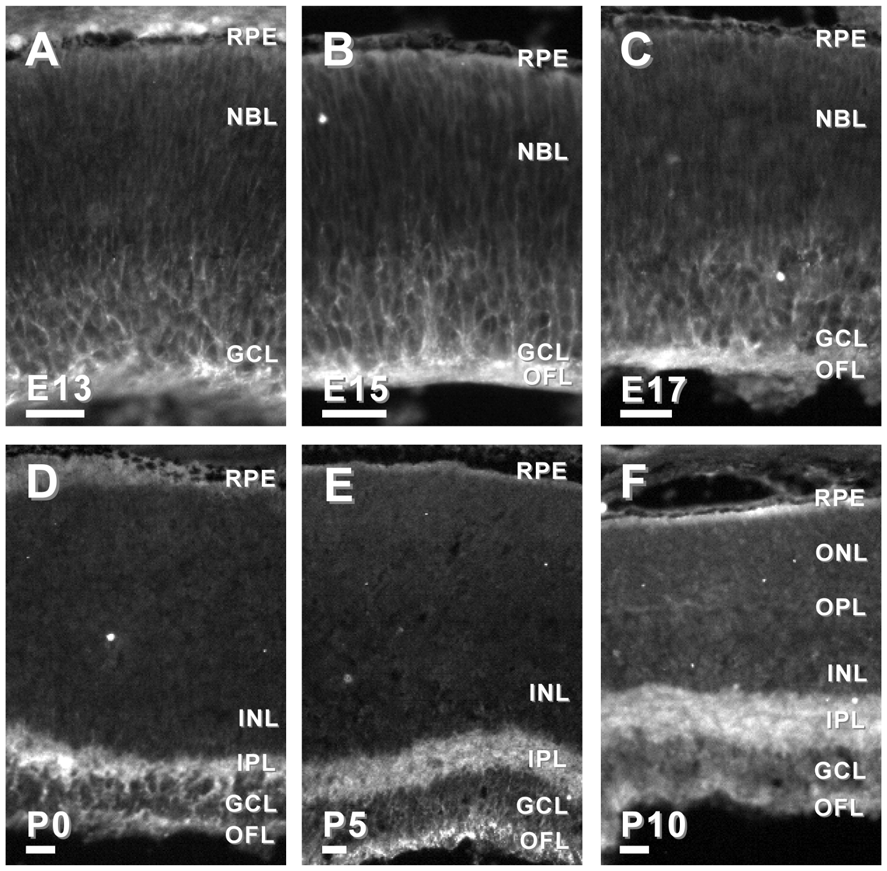
Dynamic protein expression of NDN in developing mouse retina. NDN-IR in the E13 mouse retina was localized to cells in the inner one-third of the retina (A). Similarly, in the E15 (B) and E17 (C) retinas NDN-IR was observed in the inner retina, including the GCL and OFL. In the P0 retina, NDN-IR was restricted to the developing IPL and OFL (D). Similarly, in the P5 and P10 retinas, NDN-IR remained in the IPL and OFL, respectively (E and F). Abbreviations same as in Figure 2. Bars, 30 µm.

**Figure 4:**
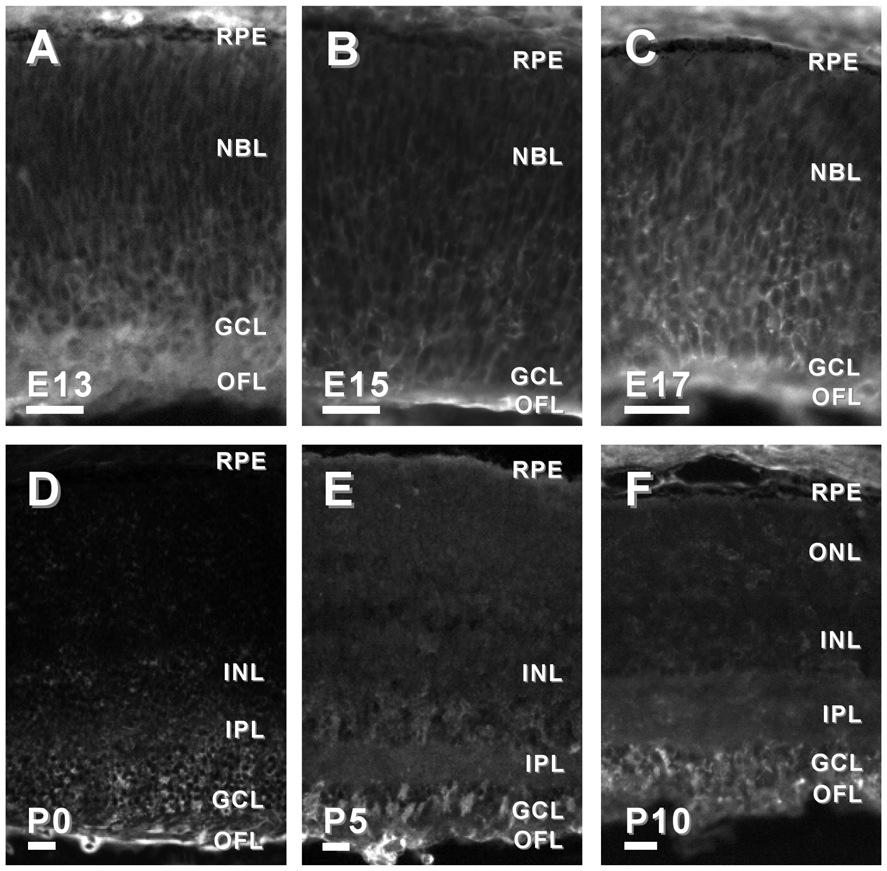
Dynamic protein expression of PAFAH1B3 in developing mouse retina. In the E13, E15 and E17 mouse retinas, PAFAH1B3-IR was observed throughout the thickness of the retina, though was slightly more intense in the cells of the inner retina (A–C). However, in the P0 retina, PAFAH1B3-IR was restricted to the GCL and OFL (D). PAFAH1B3-IR in the P5 retina was further restricted to a subset of cells in the GCL and the OFL (E). Pafah1b3-IR in the P10 retina was decreased to a punctate pattern in the GCL (F). Abbreviations same as in Figure 2. Bars, 30 µm.

**Figure 5:**
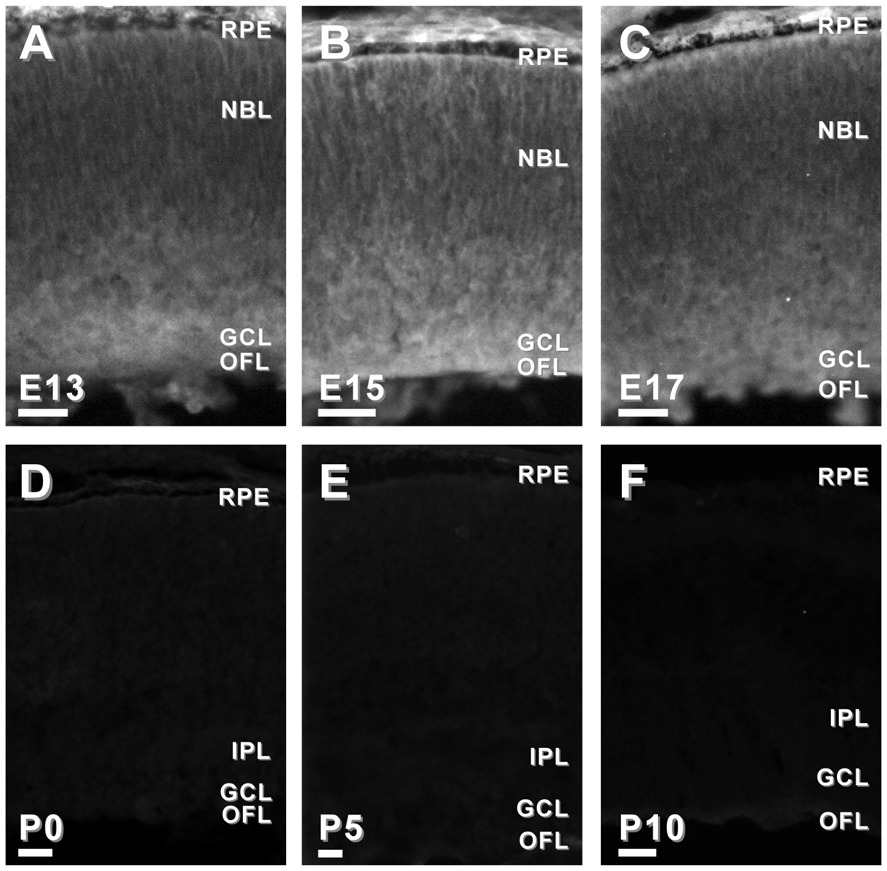
Dynamic protein expression of Psme1 in developing mouse retina. PSME1-IR in the E13 mouse retina was diffusely distributed throughout the E13, E15 and E17 retinas (A–C). However, by P0, PSME1-IR was no loner detectable above background (D). Similarly, no PSME-IR was detected in the P5 or P10 retinas (E, F). Abbreviations same as in Figure 2. Bars, 30 µm.

**Figure 6:**
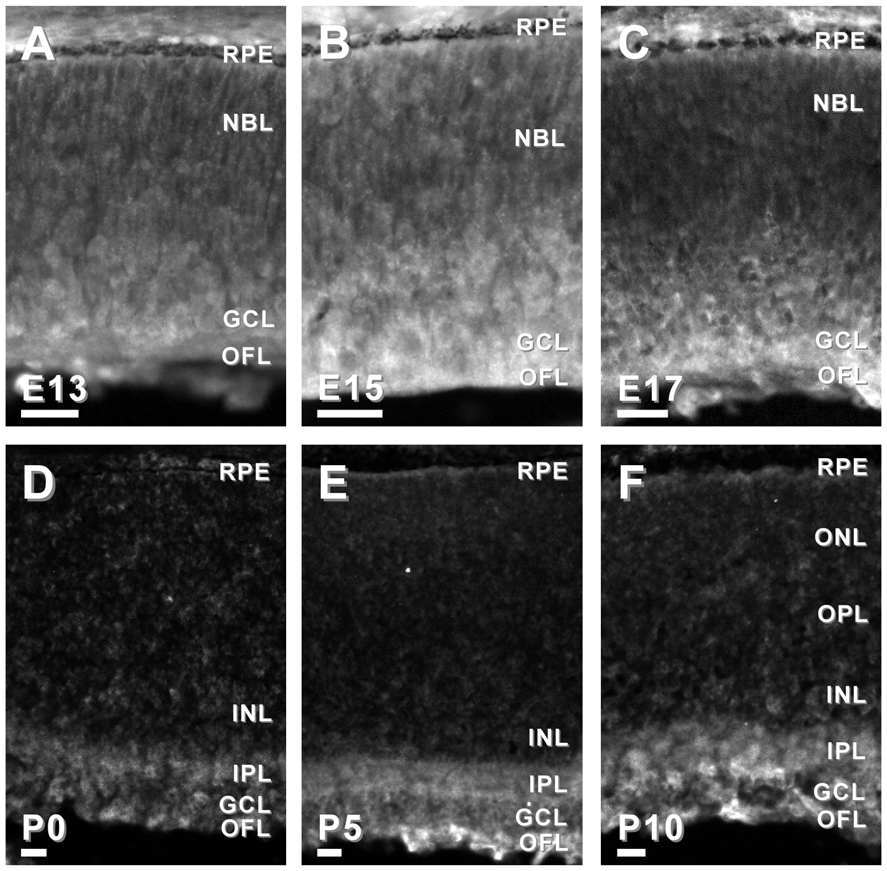
Dynamic protein expression of TSMB10 in developing mouse retina. TSMB10-IR in the E13 and E15 mouse retina was distributed throughout the retina (A, B). In the E17 mouse retina, TSMB10-IR was more intense in the inner one-third of the retina (C). By P0 TSMB10-IR in the mouse retina was largely restricted to the IPL, GCL and OFL (D). Similarly in the P5 and P10 retinas, TSMB10-IR was observed in the IPL, GCL and OFL (E, F). Abbreviations same as in Figure 2. Bars, 30 µm.

Second, we examined the spatial and temporal expression of six candidate genes in the developing mouse retina. We chose to examine candidates that had been previously reported to be associated with the developing brain, but not the developing retina, and had commercially available antibodies. Using immunohistochemistry in retinal tissue sections from mice ages embryonic day (E)13, E15, E17 and postnatal day (P)0, P5 and P10, we characterized the expression of APLP2, DPYS14, NDN, PAFAH1B3, PSME1 and TMSB10. Candidates were considered highly relevant if they were: 1) expressed in the developing retina, 2) exhibited specific (as opposed to diffuse) localization in the developing retina, and/or 3) the localization of the immunoreactivity changed as the retina matured. Based on these criteria, five of the six candidate genes tested that had not been previously associated with retinal development (*aplp2*, *ndn*, *pafah1b3*, *psme-1* and *tsmb10*) were considered good candidates for further investigation (Figures 2, 3, 4, 5 and 6).

Third, we used the biological process GO annotations Nervous System Development (0007399) and Neural Retina Development (0003407), to statistically evaluate our candidate list. In the list of 46 candidates identified by using our seed-network approach, seven of the genes had a Nervous System Development GO annotation. By using a Fisher's exact test we determined that Nervous System Development is over-represented among the group of candidate genes. The p-value for this test was 0.026, which represents the probability of seeing seven or more Nervous System Development genes in a list of 46 genes randomly selected from the 8544 genes represented on the Murine Genome U74Av2 array. Because it would be unlikely to see so many Nervous System Development genes in our candidate list of 46 genes by chance, our results suggest that Nervous System Development genes were overrepresented in our candidate list.

In summary, our analysis identified a network of 46 highly correlated candidate genes. Expression of 22 (∼47%) of these candidate genes has been previously reported in the retina or developing retina (see references in Table 3), although their specific relationship to genes within the retinal determination gene network has not been reported. We examined six candidate genes that had previously been associated with brain development, and determined that five of these genes have dynamic spatial and temporal expression in the developing mouse retina. Finally, of these 46 mammalian genes, 42 (∼91%) have homologs in fly, making them potential candidates for studies of fly retinal development as well. These findings demonstrate the powerful advantages of integrating evolutionary comparisons and systems approaches, even when approaching well-studied biological questions.

## Discussion

The compound eye of *Drosophila* is an outstanding model system to study the molecular basis of eye specification, in part, because retinal development is an organized, step-wise process with clearly demarcated regions of cell differentiation and patterning [8], [87]. These properties of the fly model have facilitated the elucidation of genetic networks involved in retinal cell differentiation and the identification of key genes required for retinal development in fly. Comparative studies between model organisms [12], [18] led to discoveries that homologous genes play important and similar roles in fly and mammalian retinal development and many of these key genes have similar connectivity in gene networks [19]. This principle of gene network conservation has motivated our development of the seed-network strategy, which we have presented here, and provides a way to validate our novel heuristic approach.

We tested our strategy using gene expression datasets from the developing mouse retina. The results from this study support our hypothesis that gene relationships in the developing fly retina are identifiable in correlation networks generated using gene expression data from the developing mouse retina. While not all gene relationships in the fly network were identified in the mouse ESN, this is not unexpected. Our results provide support for the assumption that there will be a degree of conservation within genetic networks of homologous genes, even between highly divergent species such as fly and mouse. Complete congruence between the RDGN of fly and mouse would be surprising given that these organisms possess highly divergent eye morphologies. Our results also support the second hypothesis that the mouse network derived from relationships between homologous genes from the fly RDGN (i.e. our extracted seed network [ESN]), would be an effective way to discover high quality candidate genes involved in retinal development in mouse. Our queries identified a reasonable number (46) of candidates, when compared to the hundreds or thousands of genes that correlate with a single gene of interest. The majority of our candidate genes were correlated (positively or negatively) with the same three seed genes (*Notch1*, *Eya1* and *Six3*) suggesting that these three seed genes are at the functional core of this network regulating retinal development in mouse.

At the heart of our approach is the development of biological heuristics to focus queries of relatively sparse (albeit typical) expression datasets from the developing mouse retina. It is important to note that this approach is intended to facilitate the formulation of hypotheses by providing a mechanism to integrate prior biological knowledge, but not intended to make conclusions about the function or assign significance to the candidates. The use of relationships among genes as a biological heuristic to query high-throughput data, as opposed to queries based on single genes, appears to be more profitable and efficient for the identification of additional candidates. Thus, the candidate genes we identified in this study are not end points, but are the basis of hypotheses to guide future experimental work. Traditional wet-lab experiments will be required to test these hypotheses of the specific role of each candidate gene and its placement in the gene regulatory network during mouse retinal development.

From a comparative evolutionary perspective, our results underscore the importance of looking for conservation of networks, and not just conservation at the level of individual genes. While gene orthologs may function in a similar way in a complex process or a disease state in different organisms, it is the conservation of not only the gene, but of its relationships to other genes in a network, that dramatically increases the likelihood that the gene, in fact, functions similarly. Although it has been directly demonstrated in only a few cases [19], [88]–[91], regulatory network conservation has long been the rationale for the use of model organisms to study human diseases. Comparative studies that investigate the extent of conservation in developmental regulatory networks (and of characteristics, such as modularity, connectivity, etc.) are beginning to identify common themes in networks that direct organogenesis, e.g., [92]. While it is unreasonable to expect that genetic regulatory networks controlling the development of organs in highly divergent organisms will be conserved in their entirety, application of the approach proposed here to identify conserved network modules should allow systems biologists to better capitalize on what is known in one species to advance discovery in another.

## Materials and Methods

**Figure 7:**
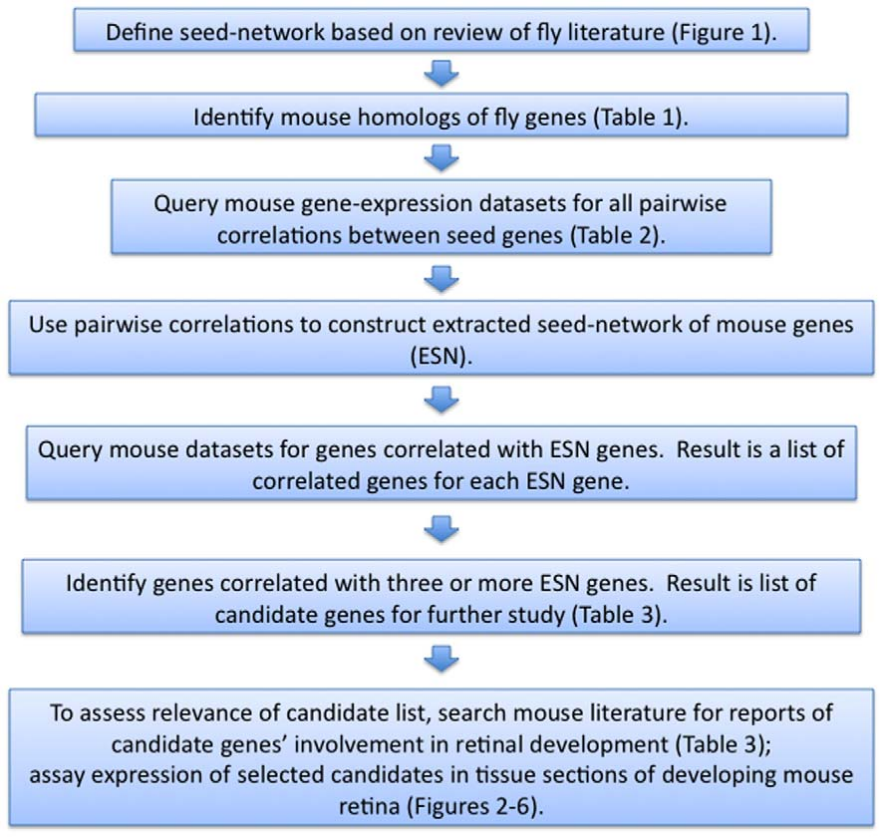
A description of our biological heuristic strategy.

Our biological heuristic strategy is described below and summarized in Figure 7.

### Construction of the fly seed network

We identified 18 genes in *Drosophila* that are involved in the retinal determination gene network (RDGN), based on published literature. Previous researchers have identified individual relationships of these genes to one another and these experimentally-determined relationships among the 18 genes were the basis of our fly seed network (see descriptions and citations in File S1).

### Homolog identification of seed network genes

Homolog identification can be difficult when comparing genomes across great evolutionary time as a result of sequence evolution and paralogous duplication events within a lineage. Because of these issues, we identified putative mouse orthologs of the *Drosophila* seed network manually, using a combination of approaches, including examination of the genomic databases FlyBase [http://flybase.org/] [93] and Mouse Genome Informatics [MGI; http://www.informatics.jax.org/] [94], phylogenetic methods presented in TreeFam [http://www.treefam.org/] [95], [96], and HomoloGene [http://www.ncbi.nlm.nih.gov/homologene] [97]. Additional assignment of orthology between fly and mouse genes was based on experimental data. For example, the mouse has three *Teashirt* (*tsh*)-like genes, *Tshz1*, *Tshz2 and Tshz3*, all of which can rescue *tsh* null mutants and induce ectopic eyes in the fly [98]. Likewise, we designated the *Pax6* isoform, *Pax6*(*5a*), found in humans and mouse, as the ortholog for fly *eyg* because the genes are structurally related [99], and we treated the mouse gene *Math5* (*Atonal7*) as the homolog to the fly gene *Atonal* based on others' work [100], [101] reviewed in [19]. Finally, qualitative and functional comparisons of the mouse genes *Six3* and *Six6* to *so* and *optix* in fly, suggest that *optix* should be treated as an ortholog of *Six3* and *Six6* [102], reviewed in [19]. Table 1 lists fly seed network genes and their mouse homolog assignment based on these data.

### Description of mouse data sets and construction of ESN

Published, freely available datasets measuring gene or protein expression in the developing mouse retina at multiple time points were collected and preprocessed as described in Hecker et al. [7]. Each dataset represents expression data collected from developing mouse retinae at multiple time points and includes: a SAGE (serial analysis of gene expression) of whole retina from Blackshaw et al. [20] was downloaded from online supplementary material; one cDNA microarray of whole retina from Zhang et al. [21] was downloaded from online supplementary material; and two Affymetrix microarrays of whole retina, the Mu74Av2 chip from Liu et al. [22] was downloaded from GEO (GDS 1845) and the Mu74Av2_1 chip from Dorrell et al. [23] was downloaded from http://www.scripps.edu/cb/friedlander/gene_expression/). These mouse datasets were designated as I, II, III, and IV, respectively, and were saved in BioNet Workbench [http://bionetworkbench.sourceforge.net/] for analysis.

We calculated Spearman Rank pairwise correlations in each mouse expression dataset using BioNet Workbench to construct the extracted seed network (ESN) for mouse. Correlation networks provide a visual representation of pairwise associations between genes in large data sets consisting of expression measurements for hundreds or thousands of genes. In a gene or protein expression correlation network, the nodes represent the genes or proteins and weighted links model interactions between them. The weight associated with a link between a pair of nodes models the correlation estimated from measurements of expression (e.g., mRNA or protein) levels of the corresponding genes across a set of experimental conditions or time points. The Spearman rank correlation measure, which assumes only an arbitrary monotonic, not necessarily linear, relationship between variables being correlated, has been demonstrated to be effective for detecting functional relationships between genes [103]. Correlation coefficients using time-course expression data are calculated by a measure of how the expression levels between any given pair of genes changes over time. Genes that are perfectly correlated with one another have a correlation coefficient of 1. Gene pairs whose expression is exactly the opposite of one another have a correlation coefficient of −1. Two genes whose expression is not correlated (no different than random) have a correlation coefficient of 0. In cases where multiple mouse paralogs for a single fly gene are present, each paralog was queried separately. Not all mouse seed genes were present in all datasets.

A link between a pair of seed network genes is supported by a dataset if the corresponding genes are positively or negatively correlated in that dataset, with the absolute value of correlation greater than or equal to 0.65 in one of the mouse datasets (I–IV). Our choice of the threshold of 0.65 for correlation was influenced by similar choices in previous studies [104]–[106] that have revealed biologically relevant links between co-expressed genes. It should be noted that we do not assign statistical significance to the value of the correlation coefficient, but rather consider the value a flexible tool that can be used at the discretion of the investigator to filter gene lists to a manageable number of candidates. This is appropriate, as our strategy is designed to facilitate the generation of hypotheses, not conclusions.

### Generating candidate gene lists

To identify candidate genes that may be involved in the gene network controlling mouse development, we used genes from the extracted seed-network (ESN; mouse homologs of fly RDGN genes whose pairwise expression correlation coefficients were >|0.65| in at least one dataset) to query large-scale gene expression datasets of the developing retina (I–IV). Each of the 17 genes from the ESN (a.k.a. seed genes) in mouse was examined separately in datasets I–IV to develop candidate gene lists. Lists were compiled by identifying genes that were correlated with individual seed genes, with a correlation coefficient greater than |0.65|. Then, gene lists for all seed genes were compared to identify candidate genes that correlated with more than one seed gene. Genes that correlated with more than three seed genes were investigated for potential biological relevance in mouse retinal development.

Biological relevance of candidate genes was assessed using manual PubMed [http://www.ncbi.nlm.nih.gov/pubmed/] [107] searches with the following search terms: retina, retinal development, CNS development, brain development, development. Gene synonymies used in the literature searches of the candidate mouse genes are listed in Table 3. Putative *Drosophila* homologs of the mouse genes from the candidate gene list were identified using FlyBase [http://flybase.org/] [93], the Mouse Genome Database (MGD) [URL: http://www.informatics.jax.org; January, 2009] [108], and TreeFam [http://www.treefam.org/] [95], [96]. When necessary, paralogous genes were included to more fully capture gene homology between the two organismal models.

In order to determine if the GO annotation Nervous System Development (0007399) was over-represented among the 46 candidate genes, a Fisher's exact test was used. Over-representation was declared if the number of genes with the GO annotation of interest on our candidate list was significantly higher than would be expected by chance, i.e., if the observed number of Nervous System Development genes was greater than would be expected when randomly selecting 46 genes from a collection of 550 Nervous System Development genes mixed with 7994 other genes. Information from version 2.4.1 of the R statistical software annotation package *mgu74av2.db* [http://www.bioconductor.org/] and release 30 of the NetAffx annotation file for the Murine Genome U74Av2 array [http://www.affymetrix.com/] was combined in order to perform the Fisher's exact test. Due to relatively frequent changes in probe set annotations and gene symbols, and also due to slight disagreements between the two annotation sources, a conservative analysis was performed. Although there are likely more genes represented on the Murine Genome U74Av2, the total number of genes represented was declared to be 8544. Similarly, the number of genes with Nervous System Development annotation was declared to be 550, although this number is likely high. Using an under-estimate of the total number of genes and an over-estimate of the genes with Nervous System Development annotation results in a higher p-value and thus more conservative results than if the true values were used.

It should also be noted that no probe set was mapped to the gene *Prrt1* in either of the current annotation sources (although it was in previous annotations), but this gene was included in the analysis because of its inclusion in the analysis of previous papers. Removing this gene from the analysis does not affect the significance of our results.

### Examining candidate gene expression with immunohistochemistry

To investigate the biological relevance of the candidate genes correlated with the ESN, we examined the spatial and temporal expression of six candidate genes in the developing mouse retina. Tissue was prepared from C57BL/6 mice in a colony maintained at Iowa State University. The gestational period of C57BL/6 mice is approximately 19 days and date of birth is designated as postnatal day 0 (P0). The developmental time series investigated included pups from embryonic days 13, 15 and 17 and postnatal days 0, 5, and 10. Mice were euthanized and their heads were removed and immersion fixed in 4% paraformaldehyde in 0.1M PO4 buffer (pH 7.5). The tissue was cryoprotected in a 30% sucrose solution in 0.1 M PO4 buffer (pH 7.4) and embedded in OCT mounting media. Tissue was sectioned at a thickness of 20 µm on a cryostat and the sections were thaw-mounted onto microscope slides and stored at −20°C. All animal procedures had the approval of the ISU committee on animal care.

Frozen tissue sections were rinsed in 0.5M KPBS and incubated in blocking solution consisting of KPBS containing 1% bovine serum albumin (Fisher, Pittsburgh, PA), 0.4% Triton-X 100 (Sigma), and 1.5% normal donkey serum (Invitrogen) for 2 hours. Cells were incubated in primary antibody overnight at 4°C. The following day slides (tissue or cells) were washed in KPBS containing 0.02% Triton-X 100 after which fluorescent secondary antibody was applied for 2 hours. After washes in KPBS containing 0.02% Triton-X 100, the slides were incubated in 300 µM DAPI diluted in KPBS. The slides were rinsed in KPBS before cover-slipping with Vectashield fluorescence mounting medium (Vector Laboratories, Burlingame, CA). The antibody sources and concentrations used for the immunohistochemical analysis are summarized in Table S1.

## Supporting Information

File S1 Seed network construction in fly.
(0.12 MB DOC)

Table S1 The antibody sources and concentrations used for the immunohistochemical analysis.
(0.04 MB DOC)
