# Supplementary material for: Using Evolutionary Conserved Modules in Gene Networks as a Strategy to Leverage High Throughput Gene Expression Queries
Source: PLoS One. 2010 Sep 2;5(9):e12525. doi: 10.1371/journal.pone.0012525 (PMC2932711; doi:10.1371/journal.pone.0012525)
Supplement: File S1 — Seed network construction in fly. (0.12 MB DOC) [file pone.0012525.s001.doc]

Supporting Information

File S1

***Seed network construction in fly***

The core of the retinal determination gene network (RDGN) in fly includes at least five genes, *twin-of-eyeless* (*toy*), *eyeless* (*ey*), *eyes absent* (*eya*), *sine oculis* (*so*), and *dachshund* (*dac*). *Ey*, and the fly paralog, *toy*, are homologs of the mammalian *Pax6* gene [12]. Work by a number of researchers has shown that *ey* is the first gene expressed in the RDGN hierarchy and is responsible for inducing the expression of *eya* and *so* [1,2,3,4]. Both *eya* and *so* participate in initiating *dac* expression [5,6]; however, it appears that feedback loops play a role in regulating expression of these genes and/or that these molecules function in one or more complexes to induce expression and initiate eye development. Both yeast two-hybrid and *in vitro* studies suggest that Eya and So and Eya and Dac, are capable of interacting with each other physically [3,5,6,7].

Both *so* and *eya* regulate the expression of the proneural gene *atonal* (*ato*), which encodes a basic-HLH protein. Eye-specific viable mutations in *so* or *eya* abolish *ato* expression [8] and work by Tanaka-Matakatsu and Du [9] have identified an eye-specific *ato* 3′ cis-regulatory sequence that is bound by So to enhance transcription. Hedgehog (Hh) also induces *ato* expression [10], as genetic studies have shown the loss of Hh and Decapentaplegic (Dpp) signal transduction prevents Ato expression [11]. Hh signaling activities are mediated by the downstream effector *Cubitus interruptus* (*Ci*), which occurs in two different forms, and the equilibrium of the two forms is controlled by Hh. Thus, Hh signaling controls initiation of retinal differentiation by releasing the *Ci*-mediated repression of *dpp* and *eya* [12].

Ectopic expression of *hh* has shown that *hh* regulates expression of *dpp* during MF progression [13,14]. Furthermore, *dpp* has a positive interaction with *eya* [15,16]. Work from Curtiss and Mlodzik [17] showed that exogeneous expression of *eya* in loss-of-function *dpp* mutant backgrounds is sufficient to rescue eye development by inducing *so* and *dac* expression. Although understanding the exact regulatory relationships between *hh* and *dpp,* and between *dpp* and *eya*, are complicated by cross-regulatory interactions [17,18,19,20,21], recent functional data suggest that *dpp* and *eya* function downstream of *hh* [12].

*Wingless* (*wg*) is linked to *homothorax* (*hth*), as Wg signaling upregulates *hth* in the eye disc [22,23,24,25]. Ectopic expression of Wg in the region anterior of morphogenetic furrow induces Hth, while blocking Wg signaling reduces Hth in the head region of the eye disc [22]. In addition, evidence indicates a link between *teashirt* (*tsh*) and *hth*: they are co-expressed during the early phase of eye disc development, and ectopic *tsh* can induce Hth and suppress eye development [25]. Furthermore, clones co-expressing *hth* and *tsh* in the eye field, retain *ey* expression and block *eya* and *dac* expression [26]. These data suggest that the Ey, Tsh, and Hth comprise a combination that prevents premature differentiation [26]. *hth* is also linked to *hairy* (*h*), and ectopic expression of Hth represses *hairy* [26], which encodes a transcriptional repressor of *ato*. Through this negative regulation of *ato*, *hairy* negatively regulates morphogenetic furrow progression and photoreceptor differentiation, as seen in a study of double mutant *h* and *extra macrochaetae* (*emc*) disc clones [27].

The genes *ocelliless* (*oc*), *hh*, and *wg* have complex and overlapping relationships in the RDGN. When *ocelliless* (*oc*) expression levels are high, *oc* regulates localization of *wg* and *hh*. *oc* mutants in the eye-antennal discs fail to terminate *wg* expression, while ectopic expression of both *wg* and *hh* activates *oc* [28,29]. In contrast, loss-of-function and complementary gain-of-function experiments of Wg and Hh signaling pathways show upregulation of *oc* [30]. These data suggest that there are several types of relationships among these genes, including autoregulation.

Many of the gene relationships described above and included in our fly seed network have been repeatedly documented and accepted for some time. We have also included, however, several recently identified or more tentative gene relationships in our seed network. For example, we recognize a relationship between *shifted* (*shf*) and *ey*, which was initially identified by microarray analysis and confirmed through *in vitro* and *in vivo* methods by Ostrin et al. [31]. We also recognize a hypothesized relationship between *optix* and *ato* (meaning other genes may be in-between *optix* and *ato*), based on expression patterns and comparative evidence in fly (see review in [32]). Furthermore, electrophoretic mobility shift assays and reporter gene analysis suggests *optix* is activated by Ey [31]. In addition, we indicate a relationship between *Notch* (*N*) and the *Pax* gene member *eye gone* (*eyg*) [33]. While not directly linked to our fly seed network, data from *Drosophila* [34,35] demonstrate that these two genes are important for eye growth rather than eye differentiation; overexpression of Eyg rescues *Notch* mutants to regulate eye growth.

**References**

1. Quiring R, Walldorf U, Kloter U, Gehring WJ (1994) Homology of the *eyeless* gene of Drosophila to the *small eye* gene in mice and *aniridia* in humans. Science 265: 785-789.

2. Halder G, Callaerts P, Gehring WJ (1995) Induction of ectopic eyes by targeted expression of the *eyeless* gene in *Drosophila*. Science 267: 1788-1792.

3. Bonini NM, Bui QT, Gray-Board GL, Warrick JM (1997) The *Drosophila* *eyes* *absent* gene directs ectopic eye formation in a pathway conserved between flies and vertebrates. Development 124: 4819-4826.

4. Niimi T, Seimiya M, Kloter U, Flister S, Gehring WJ (1999) Direct regulatory interaction of the eyeless protein with an eye-specific enhancer in the sine oculis gene during eye induction in *Drosophila*. Development 126: 2253-2260.

5. Pignoni F, Hu B, Zavitz KH, Xiao J, Garrity PA, et al. (1997) The eye-specification proteins So and Eya form a complex and regulate multiple steps in *Drosophila* eye development. Cell 91: 881-891.

6. Chen R, Amoui M, Zhang Z, Mardon G (1997) Dachshund and eyes absent proteins form a complex and function synergistically to induce ectopic eye development in Drosophila. Cell 91: 893-903.

7. Chen F, Figueroa DJ, Marmorstein AD, Zhang Q, Petrukhin K, et al. (1999) Retina-specific nuclear receptor: a potential regulator of cellular retinaldehyde-binding protein expressed in retinal pigment epithelium and Muller glial cells. PNAS 96: 15149-15154.

8. Jarman AP, Grell EH, Ackerman L, Jan LY, Jan YN (1994) Atonal is the proneural gene for Drosophila photoreceptor. Nature 369: 398-400.

9. Tanaka-Matakatsu M, Du W (2008) Direct control of the proneural gene *atonal* by retinal determination factors during *Drosophila* eye development. Developmental Biology 313: 787-801.

10. Dominguez M, Hafen E (1997) Hedgehog directly controls initiation and propagation of retinal differentiation in the *Drosophila* eye. Genes & Development 11.

11. Greenwood S, Struhl G (1999) Progression of the morphogenetic furrow in the *Drosophila* eye: the roles of Hedgehog, Decapentaplegic and the Raf pathway. Development 126: 5795-5808.

12. Pappu KS, Chen R, Middlebrooks BW, Woo C, Heberlein U, et al. (2003) Mechanism of hedgehog signaling during *Drosophila* eye development. Development 130: 3053-3062.

13. Heberlein U, Singh CM, Luk AY, Donohoe TJ (1995) Growth and differentiation in the Drosophila eye coordinated by hedgehog. Nature 373: 709-711.

14. Heberlein U, Wolff T, Rubin GM (1993) The TGF beta homolog dpp and the segment polarity gene hedgehog and required for propagation of a norphogenetic wave in the Drosophila retina. Cell 75: 913-926.

15. Bessa J, Casares F (2005) Restricted *teashirt* expression confers eye-specific responsiveness to Dpp and Wg signals during eye specification in *Drosophila*. Development 132: 5011-5020.

16. Halder G, Callaerts P, Flister S, Walldorf U, Kloter U, et al. (1998) Eyeless initiates the expression of both sine oculis and eyes absent during *Drosophila* compound eye development. Development 125: 2181-2191.

17. Curtiss J, Mlodzik M (2000) Morphogenetic furrow initiation and progression during eye development in *Drosophila*: the roles of *decapentaplegic*, *hedgehog* and *eyes absent*. Development 127: 1325-1336.

18. Borod ER, Heberlein U (1998) Mutual regulation of *decapentaplegic* and *hedgehog* during the initiation of differentiation in the *Drosophila* retina. Developmental Biology 197: 187-197.

19. Chen R, Halder G, Zhang Z, Mardon G (1999) Signaling by the TGF-beta homolog *decapentaplegic* functions reiteratvely within the network of genes controlling retinal cell fate determination in *Drosophila*. Development 126: 935-943.

20. Hazelett DJ, Bourouis M, Walldorf U, Treisman JE (1998) *decapentaplegic* and *wingless* are regulated by *eyes absent* and *eyegone* and interact to direct the pattern of retinal differentiation in the eye disc. Development 125: 3741-3751.

21. Pignoni F, Zipursky SL (1997) Induction of *Drosophila* eye development by *decapentaplegic*. Development 124: 271-278.

22. Pichaud F, Casares F (2000) *homothorax* and *iroquois-C* genes are required for the establishment of territories within the developing eye disc. Mechanisms of Development 96: 15-25.

23. Lee JD, Treisman JE (2001) The role of Wingless signaling in establishing the anteroposterior and dorsoventral axes of the eye disc. Development 128: 1519-1529.

24. Baonza A, Freeman M (2002) Control of Drosophila eye specification by Wingless signalling. Development 129: 5313-5322.

25. Singh A, Kango-Singh H, Sun YH (2002) Eye suppression, a novel function of teashirt, requires Wingless signaling. Development 129: 4271-4280.

26. Bessa J, Gebelein B, Pichaud F, Casares F, Mann RS (2002) Combinatorial control of *Drosophila* eye development by Eyeless, Homothorax, and Teashirt. Genes & Development 16: 2415-2427.

27. Brown NL, Sattler CA, Paddock SW, Carroll SB (1995) Hairy and Emc negatively regulate morphogenetic furrow progression in the *Drosophila* eye. Cell 80: 879-887.

28. Royet J, Finkelstein R (1996) hedgehog, wingless and orthodenticle specify adult head development in *Drosophila*. Development 122: 1849-1858.

29. Royet J, Finkelstein R (1997) Establishing primordia in the *Drosophila* eye-antennal imaginal disc: the roles of *decapentaplegic*, *wingless* and *hedgehog*. Development 124: 4793-4800.

30. Blanco J, Seimiya M, Pauli T, Reichert H, Gehring WJ (2009) Wingless and Hedgehog signaling pathways regulate *orthodenticle* and *eyes* *absent* during ocelli development in *Drosophila*. Developmental Biology 329: 104-115.

31. Ostrin EJ, Li Y, Hoffman K, Liu J, Wang K, et al. (2006) Genome-wide identification of direct targets of the *Drosophila* retinal determination protein Eyeless. Genome Research 16: 466-476.

32. Friedrich M (2006) Ancient mechanisms of visual sense organ development based on comparison of gene networks controlling larval eye, ocellus, and compound eye specification in *Drosophila*. Arthropod Structure and Development 35: 357-378.

33. Yao JG, Sun YH (2005) Eyg and Ey Pax proteins act by distinct transcriptional mechanisms in *Drosophila* development. EMBO Journal 24: 2602-2612.

34. Dominguez M, Ferres-Marco D, Gutierrez-Avino, Speicher SA, Beneyto M (2004) Growth and specification of the eye are controlled independently by Eyegone and Eyeless in *Drosophila melanogaster*. Nature Genetics 36: 31-39.

35. Chao JL, Tsai YC, Chiu SJ, Sun YH (2004) Localized Notch signal acts through eyg and upd to promote global growth in *Drosophila* eye Development 131: 3839-3847.
