# Supplementary material for: Using Evolutionary Conserved Modules in Gene Networks as a Strategy to Leverage High Throughput Gene Expression Queries
Source: PLoS One. 2010 Sep 2;5(9):e12525. doi: 10.1371/journal.pone.0012525 (PMC2932711; doi:10.1371/journal.pone.0012525)
Supplement: Table S1 — The antibody sources and concentrations used for the immunohistochemical analysis. (0.04 MB DOC) [file pone.0012525.s002.doc]

**Table S1:** The antibody sources and concentrations used for the immunohistochemical analysis

| Antibody | Species Produced In | Vendor | Concentration |
| --- | --- | --- | --- |
| **Primary Antibodies** | | | |
| Aplp2 | Rabbit | Abcam | 1:500 |
| Dpys14 (crmp3) | Rabbit | Abcam | 1:100 |
| Ndn | Goat | Santa Cruz | 1:10 |
| Pafah 1b3 | Mouse | Santa Cruz | 1:500 |
| Psme1 | Rabbit | Protein tech Group | 1:10 |
| Tmsb10 | Rabbit | Santa Cruz | 1:10 |
| **Secondary Antibodies** | | | |
|  |  |  |  |
| Donkey-anti-Rabbit Alexa Fluor 594 | Molecular Probes |  | 1:500 |
| Donkey-anti-Mouse Alexa Fluor 594 | Molecular Probes |  | 1:500 |
| Donkey-anti-Goat Alexa Fluor 594 | Molecular Probes |  | 1:500 |
